# Long-Term Sweat Testing Dataset for Second-Life Batteries

**DOI:** 10.1038/s41597-025-05360-7

**Published:** 2025-06-23

**Authors:** Matthew Beatty, Dani Strickland, Joe Warren, John Chan, Pedro Ferreira

**Affiliations:** 1 Wolfson School of Mechanical, Electrical and Manufacturing Engineering, Loughborough University, Loughborough, LE11 3TU UK; 2 Powervault, Unit 9, Garrick Industrial Centre, Irving Way, London, NW9 6AQ UK

**Keywords:** Electrical and electronic engineering, Energy grids and networks

## Abstract

This paper describes a long-term cycling dataset of repurposed lithium-ion batteries originally used in electric vehicles. After their initial automotive use – referred to as their “first life” – these batteries were redeployed for stationary energy storage applications, representing their “second life”. The dataset covers six distinct use cases modelled to represent real-world energy storage applications. Unlike other published datasets, which focus on new or first-life cells, this work exclusively features second-life batteries with no available data from prior usage. It is aimed at supporting research into battery degradation, state of health prediction, and performance benchmarking under aged conditions. Data was collected using a Chroma 17020 batter cycler and originally stored as large, continuous log files. This paper outlines the steps taken to reorganise and clean the data – handling missing values and segmenting it into individual cycles – while preserving its raw experimental content. Cycling occurred intermittently between 2019 and 2025, offering a uniquely long observation period. The dataset supports both academic and industrial research into battery ageing and second-life applications.

## Background & Summary

Lithium-ion batteries are widely used across various applications, including consumer electronics, electric vehicles (EVs), domestic energy storage systems (ESS) and grid-scale ESS’s^1^. As the adoption of EVs has grown, this has in turn resulted in an increasing number of EV batteries reaching the end of their first life within vehicles^2–4^. However, these batteries are typically retired when they have around 70–80% of their original capacity remaining, making them unsuitable for automotive use, but still viable for less demanding second-life applications. Instead of being immediately recycled, they can be repurposed for uses such as grid support, renewable energy storage, and backup power solutions, extending their overall usability and reducing waste^5–7^.

There is extensive research on tracking degradation and capacity fade in new, first-life lithium-ion batteries. These studies are mainly conducted under controlled laboratory conditions, using consistent charge-discharge patterns, and full depth of discharge (DOD) cycles. Publicly available datasets also focus on small-capacity cells (≤2 Ah), which are cheaper to test in bulk, but do not reflect real-world operational conditions. These datasets commonly assume a battery starts at full capacity and degrades predictably to an end-of-life (EOL) threshold of 80% capacity^8,9^. While this has enabled the development of various state of health (SOH) estimation models, they are largely tailored to idealised scenarios involving new batteries.

In contrast, second-life batteries (SLBs), which are repurposed after their initial use in electric vehicles, often enter service with an already reduced and unknown capacity. Their degradation trajectories are shaped by their first-life usage patterns, cycling histories, and varying DOD levels, which are often undocumented. This makes it difficult to apply traditional SOH estimation methods directly. Although SLBs may still retain 70–80% of their original capacity and are viable for stationary applications such as grid support, backup power, and renewable energy storage, there are very few publicly available long-term cycling datasets that reflect the potential of second-life usage.

A key limitation in SLB research is the lack of standardised and diverse datasets. In practice, SLBs are sourced from a wide range of EV manufacturers, and come with varying chemistries, capacities, size, and shapes. To address this, there is a growing need for datasets that reflect the full range of SLB characteristics and usage histories. By expanding the diversity and scale of available data, more robust and generalisable models for SOH estimation and degradation can be developed. Table 1 below provides an overview of some publicly available datasets with details of the number of cells, the nominal capacity, cell chemistry, test conditions, and the lowest recorded state of health (SOH):

**Table 1:** Overview of publicly available battery datasets.

Reference Study	Test Conditions	Cell Information
NASA^16,17^	Charge/discharge cycling at different temperatures; deep discharge cycles to 2.7 V and below; end-of-life defined at 30% capacity fade.	34 cells; 2.0 Ah; LCO (LiCoO_2_); SOH: 100-70%
Oxford^13^	40 °C thermal chamber; CC-CV charging; Urban Artemis profile drive cycle discharge; Characterization every 100 cycles.	8 cells; 0.74 Ah, LCO (LiCoO_2_); SOH: 100-75%
CALCE^18^: INR 18650-20 R	Low-current and incremental OCV-SOC tests; Three temperatures tested; Four dynamic stress tests for SOC tracking.	48 cells; 2.0 Ah; LiNiMnCo; SOH: 100-60%
CALCE^18^: CS2 Battery	Cycled at multiple current rates (0.5 C, 1 C, pulsed currents); Some cells had varied cut-off voltages.	15 cells; 1.1 Ah; LiCoO_2_ cathode; SOH: 100-60%
CALCE^18^: CX2 Battery	Multiple current cycling tests (0.5 C, 1 C, 3 C, pulsed); Some cycled under temperature variations (25 °C–55 °C).	12 cells; 1.35 Ah; LiCoO_2_ cathode; SOH: 100-60%
CALCE^18^: PL (Pouch Cells)	Partial charge-discharge cycling under different SOC limits (e.g., 20%-80%, 0%-60%); Some cells cycled with 2 C discharges.	16 cells; 1.5 Ah; LiCoO_2_ cathode; SOH: 100-60%
Severson (Data-driven prediction)^14^	Fast charging with one-step/two-step CC policies; 30 °C temperature chamber; 4 C discharges; EOL defined at 80% capacity retention.	124 cells; 1.1 Ah; LiFePO4; SOH: 100-75%
Stroebl *et al*. (Multi-stage aging)^19^	Multi-stage aging protocols with accelerated cycling, rest periods, and variable temperatures. 93 different aging conditions.	279 cells; 1.1-2.0 Ah; NMC/SiOx; SOH: 100-60%
Luh & Blank (Comprehensive battery aging)^20^	Standard CC-CV cycling with periodic impedance and capacity measurements under controlled temperatures.	228 cells; 1.5-2.0 Ah; NMC/C-Si; SOH: 100-50%
Long-Term Sweat Testing SLBs (this paper)	6 conditions: long-term cycling based off of real-word energy storage systems.	6 modules (2S2P); 66 Ah; LMO/LNO; SOH: 70-30%

The primary aim of this paper is to provide researchers with access to detailed, long-term cycling data from SLBs operating in realistic application scenarios. Given the limited availability of such datasets, particularly for batteries beyond their first life, this work supports efforts in SOH estimation, degradation analysis, and the evaluation of SLB performance across varied operating conditions. The dataset is particularly suited for developing and validating predictive models and guiding operational strategies for repurposed energy storage.

This dataset delivers on the need to address gaps in test data for larger cells and second life application by providing long-term cycling data for SLBs across six different use cases. Each use case represents a unique operational scenario, incorporating variations in charge-discharge profiles, rest periods, and cycling intensities. Six SLBs were tested, each corresponding to a specific use case, allowing for a broad range of experimental conditions. Capacity tests were performed periodically, with full capacity values recorded at the beginning and end of each use case. The dataset was recorded at a sampling rate of approximately every 2.8 seconds, offering high-resolution insights into performance trends and degradation over time.

This dataset has also been utilized in other published studies. The first study set the foundation for this dataset by developing the experimental framework for testing these SLBs^10^ – “Sweat Testing Cycles of Batteries for Different Electrical Power Applications”. This study explored six different use cases for a domestically located battery system and used MATLAB to generate charge and discharge profile that represented real-world operating conditions over a year-long period. Statistical analysis methods, including the Haar transform and a pragmatic approach, were applied to condense these waveforms into programmable steps, forming the basis for the cycling protocols used in this dataset^10^.

Further analysis using this dataset was conducted in the following research paper^11^ - “Second-Life Battery Capacity Estimation and Method Comparison”. This research investigated the effectiveness of different heuristic state-of-health (SOH) estimation methods originally designed for first-life capacity estimations. The study evaluated various algorithms to assess their suitability for forecasting battery capacity fade in second-life applications. The findings highlighted that a modified Gaussian regression technique could provide a practical and reliable approach for estimating battery capacity in SLBs.

Finally, this dataset was also used in the development of a standardized methodology for generating incremental capacity curves^12^ – “A Review of Methods of Generating Incremental Capacity-Differential Voltage Curves for Battery Health Determination”. The proposed method was validated using three datasets: two publicly available datasets^13,14^, and one of the use cases from this dataset. By establishing a consistent approach to deriving incremental capacity curves, this study aimed to improve the reliability and repeatability of SOH assessments for Li-ion batteries. These prior studies demonstrate the broad application of this dataset and its potential to support further research into second-life battery performance especially in the field of AI and machine learning approaches to degradation.

## Sweat Testing Methodology

The battery cycling tests were conducted using a Chroma 17020 battery cycler, which served as the central platform for programmable charge-discharge operations. The raw data were logged by the Chroma system and saved as.DB files, which were later extracted and converted into.csv format. Each.csv file corresponds to a single cycle and contains key parameters, including voltage, current, charge/discharge duration, and temperature.

**Fig. 1:**
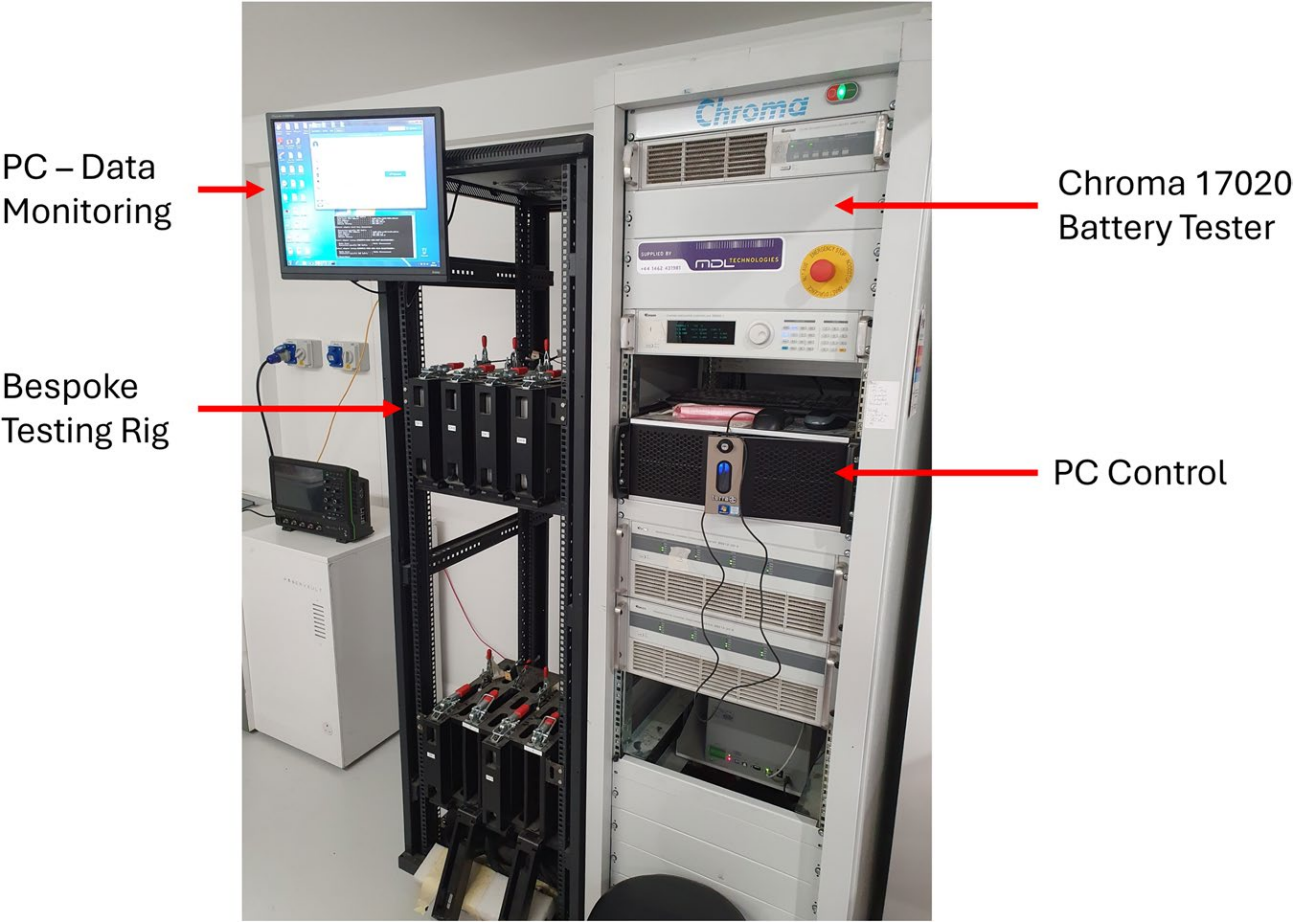
Experimental setup for the sweat test cycling using a Chroma 17020 battery cycler.

Figure 1 shows the Chroma 17020 system, comprising multiple test channels capable of parallel cycling. Each channel is controlled via a central PC, allowing real-time acquisition and management of test parameters. The cleaning and processing steps for the data extraction is further explained afterwards.

**Fig. 2:**
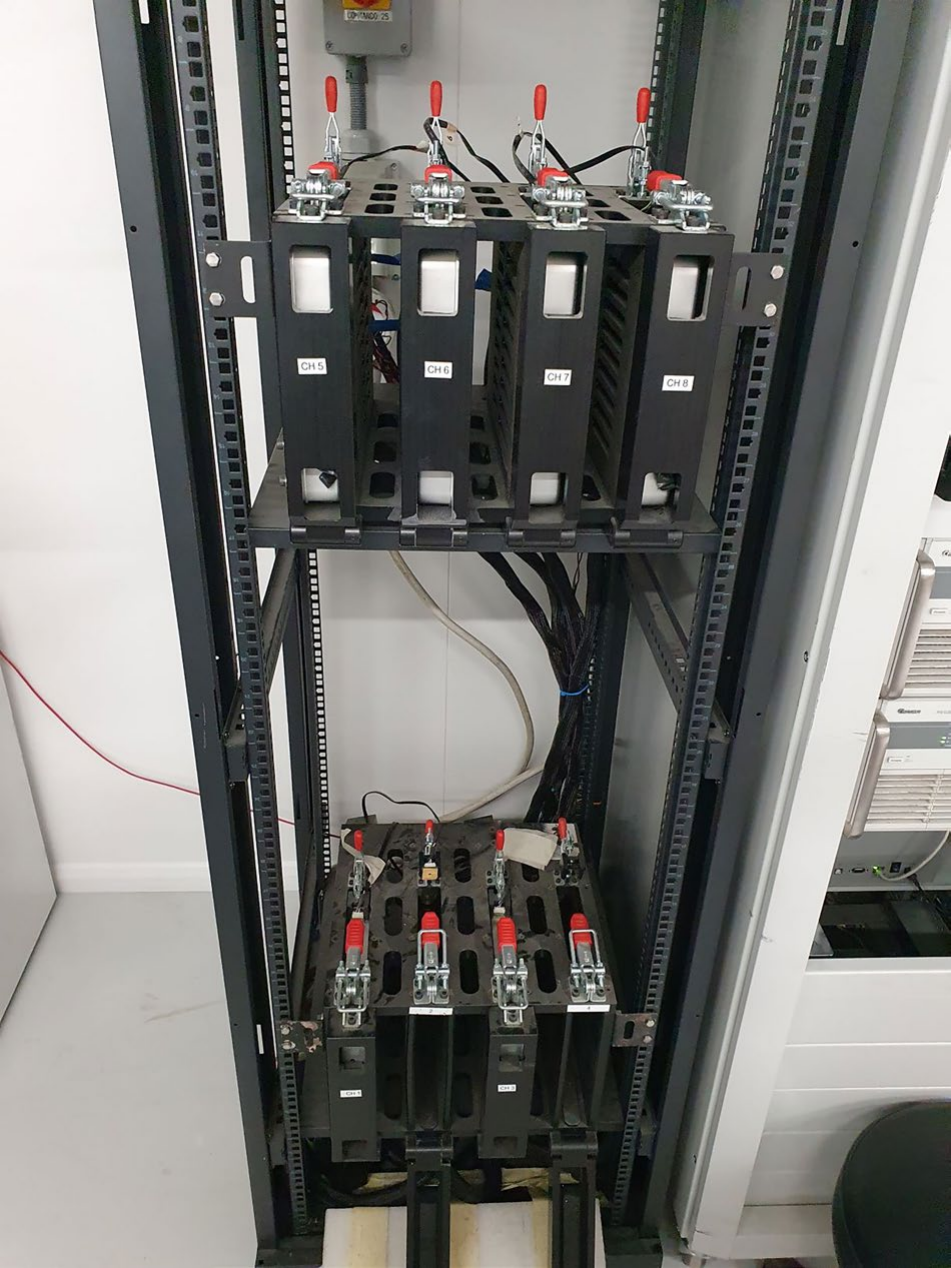
Chroma bespoke test rig for loading batteries.

Each test channel was configured with an individual battery module houses within a custom-designed mechanical rig. The rig securely clamps the modules in place, ensuring a stable electrical connection throughout the testing period. Figure 2 shows a closer view of this fixture, where current and voltage leads were directly attached to the positive and negative terminals of each module.

These electrical connections were interfaced with the power condition units (for executing the charging-discharging routines) and the data acquisition system (for recording voltage, current, and auxiliary measurements), both integral to the Chroma tester. A central PC Current running the Chroma software controlled the test profiles, logged the data, and managed channel-level communication. Figure 1 also highlights this interface, including the screen showing active cycling parameters.

**Fig. 3:**
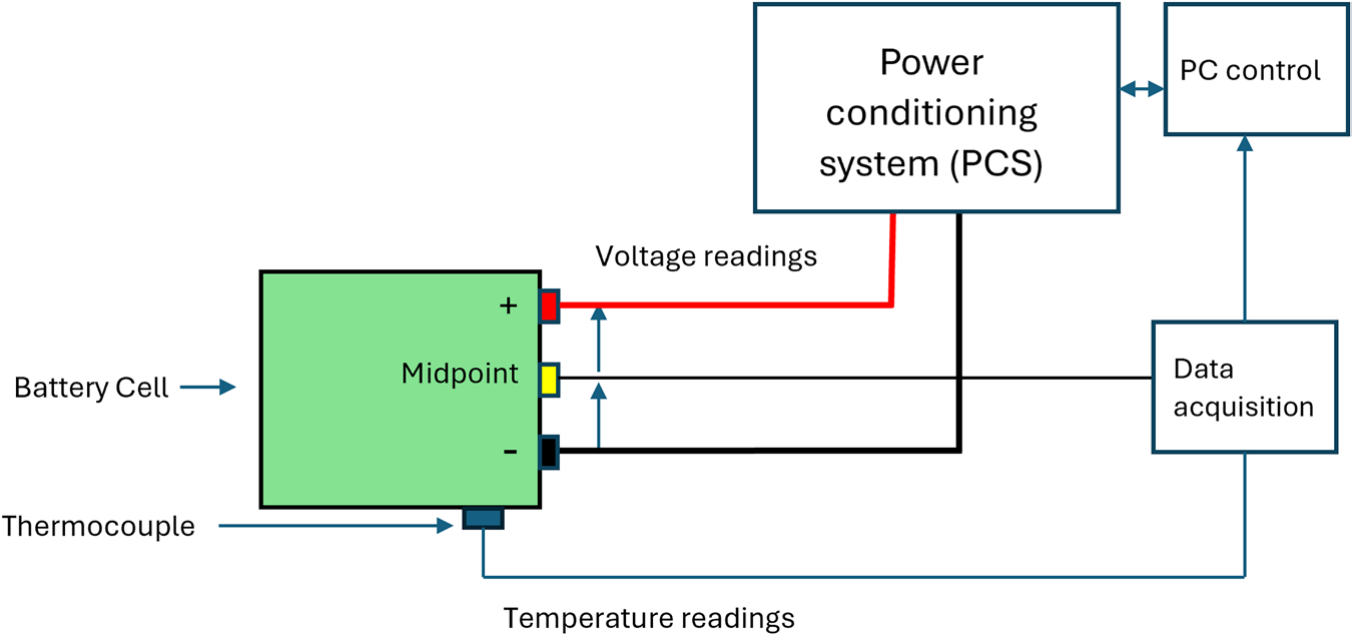
Illustration of a singular channel of the Chroma system for testing the SLBs.

Figure 3 presents a schematic representation of a single test channel. This includes voltage and current sensing wires connected directly to the battery terminals, as well as thermocouples paced on the side of each module to capture surface temperature data. The sensors and power conditioning circuity are routed back to the Chroma’s internal monitoring system and then to the central control PC for logging and feedback control. Each channel in the multi-channel setup follows the same configuration.

The batteries tested were retired lithium-ion modules previously used in an EV. Each module consists of two pouch cells in series and two in parallel (2S2P configuration). As the first-life history of these modules is not available, all data presented in this work was collected from the beginning of their second-life usage.

Key specifications of the modules are as follows:

- Cell chemistry – Lithium Manganese Oxide (LMO) and Lithium Nickel Oxide (LNO)
- Nominal capacity (new) – 66 Ah
- Minimum voltage – 5 V per module
- Nominal voltage – 7.5 V per module
- Maximum voltage – 8.3 V per module
- Dimensions: 303 mm (Length) × 223 mm (Width) × 35 mm (Height)
- Weight: 3.8 kg

**Fig. 4:**
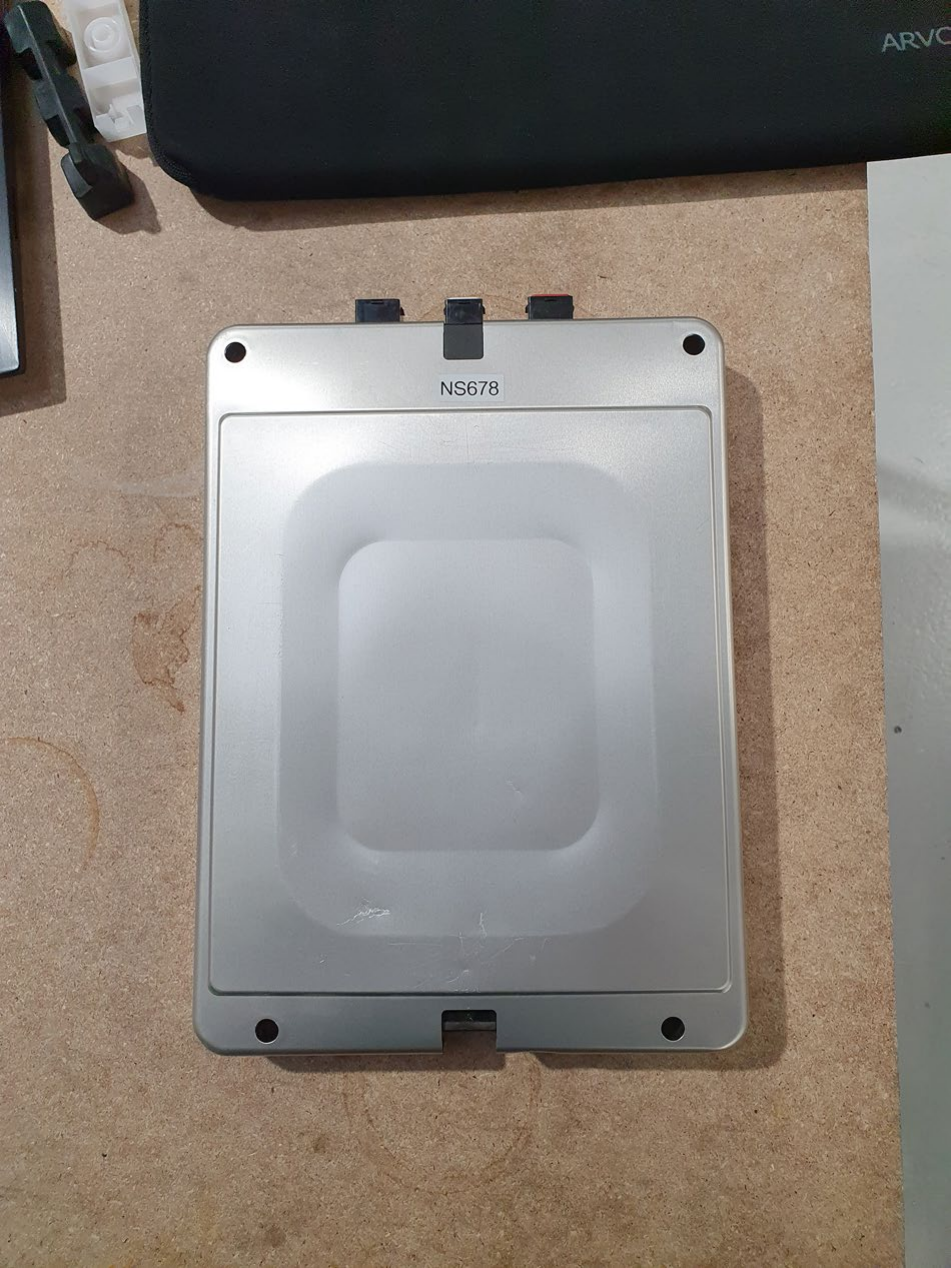
Plan view image of the second-life lithium-ion modules being tested.

**Fig. 5:**
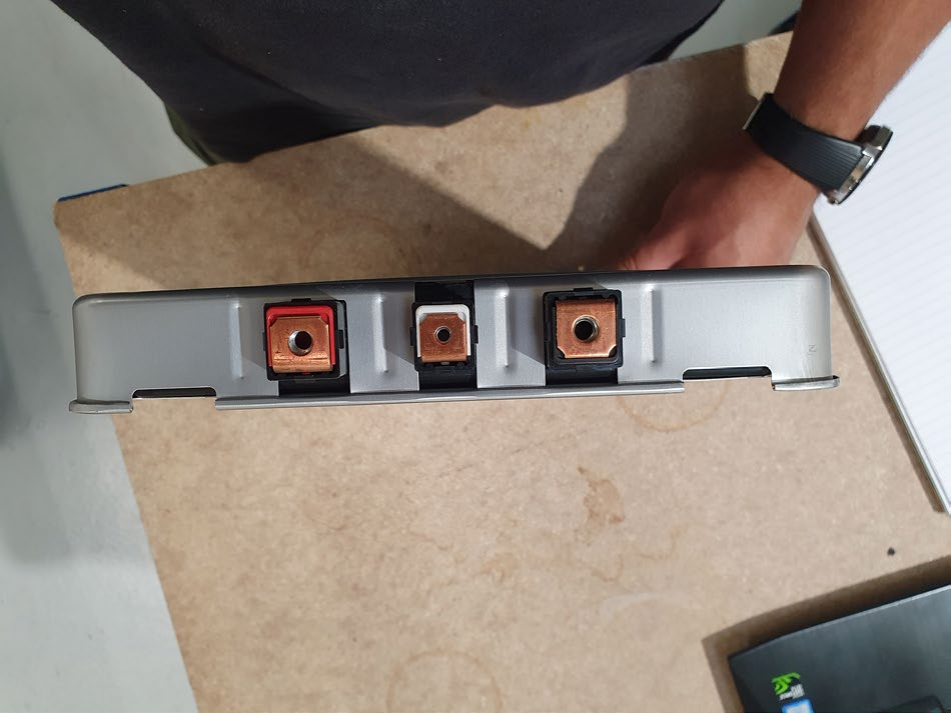
Plan view of the terminals of the second-life lithium-ion modules being tested.

Figs. 4 and 5 display images of the SLB modules tested in this study, with Fig. 5 specifically highlighting the positive and negative terminals of a singular module.

The term “sweat testing” refers to a method for replicating battery usage profiles over extended periods, enabling accelerated evaluation of battery behaviour under practical operating conditions. In this study, testing was conducted in a factory environment rather than a temperature-controlled laboratory, providing a closer approximation to real-world use. As a result, ambient temperature was not strictly regulated, and the SLBs were exposed to natural room temperature fluctuations across seasons. These ambient temperatures were recorded via the thermocouples attached to each module, and the corresponding temperature data is included in the dataset.

Rather than applying simple, uniform cycling protocols, sweat testing involves encoding diverse, real-world energy use profiles into programmable charge-discharge sequences. This allows for the simulation of operational stresses from applications such as residential storage, grid-services, and market trading, which helps capture the long-term effects of irregular cycling and varying load intensities under ambient environmental conditions.

The dataset includes six distinct use cases, each designed to simulate different real-world applications. More details of each use can be found in the previous research paper^10^.

1. PV: A house with four people and a photovoltaic (PV) panel using the battery module to absorb excess energy when the PV panel generates more power than is consumed in the house. The battery module then releases this stored energy when needed.
2. PV-TOU: A house with four people and PV panels operating a time-of-use (TOU) tariff, where energy is stored and discharged based on tariff fluctuations.
3. TOU: A house with four people but no PV, where the battery module charges during low-tariff periods and discharges during peak tariff times to reduce electricity costs.
4. FFR: The battery participates in an aggregated firm frequency response (FFR) system, charging and discharging dynamically in response to grid frequency fluctuations.
5. EFR: Similar to FFR but with more rapid cycling, optimizing the battery module for performance on the enhance frequency response (EFR) market.
6. Day Ahead: The battery module is used as part of an aggregated system to trade energy in the day-ahead electricity market, charging when prices are low and discharging when prices increase.

The first three use cases follow a quarterly cycle, with each quarter having a different number of charge-discharge steps, whilst the last three use cases following a year-long cycling profile. The charge-discharge sequences consist of constant current (CC) discharges, constant-current constant-voltage (CC-CV) charges, and intermittent rest periods. Each ‘step’ in the testing profile equates to a sequence of charge, discharge, or a rest period. A brief overview of each testing profile can be found in Table 2 below, whilst more details of each step can be found in the supplementary information.

**Table 2:** Overview of profile descriptions for each use case.

Use Case	Testing Period	Total Steps	Profile Description
PV – Q1	January-March	43	Alternating CC discharge and CC-CV charge with periodic rest
PV – Q2	April-June	57	Increased CC-CV charge cycles compared to Q1
PV – Q3	July-September	41	Similar to Q1, slight variations in rest periods
PV – Q4	October-December	29	Reduced CC-CV charging and fewer total steps
PV-TOU – Q1	January-March	37	Similar to PV but optimized for TOU tariffs with periodic rest
PV-TOU – Q2	April-June	39	Minimal variations from Q1, slightly increased discharge cycles
PV-TOU – Q3	July-September	39	Same as Q2, maintains consistent cycling pattern
PV-TOU – Q4	October-December	39	Similar to Q2 and Q3, minor differences in rest intervals
TOU – Q1	January-March	35	Primarily CC discharge with CC-CV charge and periodic rest
TOU – Q2	April-June	34	Reduced total steps compared to Q1, minor variations in rest
TOU – Q3	July-September	30	Further reduction in CC-CV charge cycles
TOU – Q4	October-December	29	Lowest step count, optimized for end-of-year consumption trends
FFR	Year-long	48	Frequent cycling for grid frequency response, alternating CC discharge and CC-CV charge
EFR	Year-long	143	High-frequency response cycling, rapid alternation between CC discharge and CC-CV charge
Day Ahead	Year-long	85	Market-driven cycling strategy based on energy price signals, balancing charge and discharge cycles

The sweat testing methodology was designed to generate representative charge-discharge waveforms for each use case based on their real-world cycling behaviour as outlined in prior work^10^. The development of these testing waveforms, including the statistical method used to encode them, was originally presented in the previous work^10^ and is summarised here to support understanding of the testing procedures used to generate this dataset.

The waveforms were extracted from MATLAB-generated charge-discharge profiles and refined to eliminate inactive periods, ensuring that the resulting dataset only reflected active battery operation.

To convert these realistic-use case waveforms into programmable test sequences, two transformation techniques were employed. The Haar wavelet transform allowed for efficient signal compression and the regeneration of transient cycle behaviour, while a statistical averaging method provided an alternative approach for battery tested that do not support high-speed transitions. Both techniques preserved the variability and stress characteristics of real-world use cases, enabling robust evaluation of SLB degradation under different operational scenarios.

The two methods are summarised as follows:

1. Haar wavelet transformation
  The Haar transform was used decompose the charge-discharge waveforms into a sequence of square-shaped functions. This is similar to a Fourier transform but is more optimised for capturing discrete transitions in cycling behaviour. This method is suitable for battery tested capable of high-speed cycling transitions. The resulting Haar coefficients used to extract key cycling components, which were then recombined to reconstruct a simplified but representative charge-discharge pattern that closely matched the original waveform.
2. Statistical averaging
  As not all battery testing equipment can support high-frequency transitions, an alternative approach was also developed. This method involved statistically analysing the total time the battery operated at different charge and discharge rates over a given period. These distributions were plotted as histograms, which were then averaged to generate a representative daily cycle for that month. This approach allowed for an approximate but practical reconstruction of the charge-discharge behaviour whilst maintaining consistency with the original cycling conditions.

**Fig. 6:**
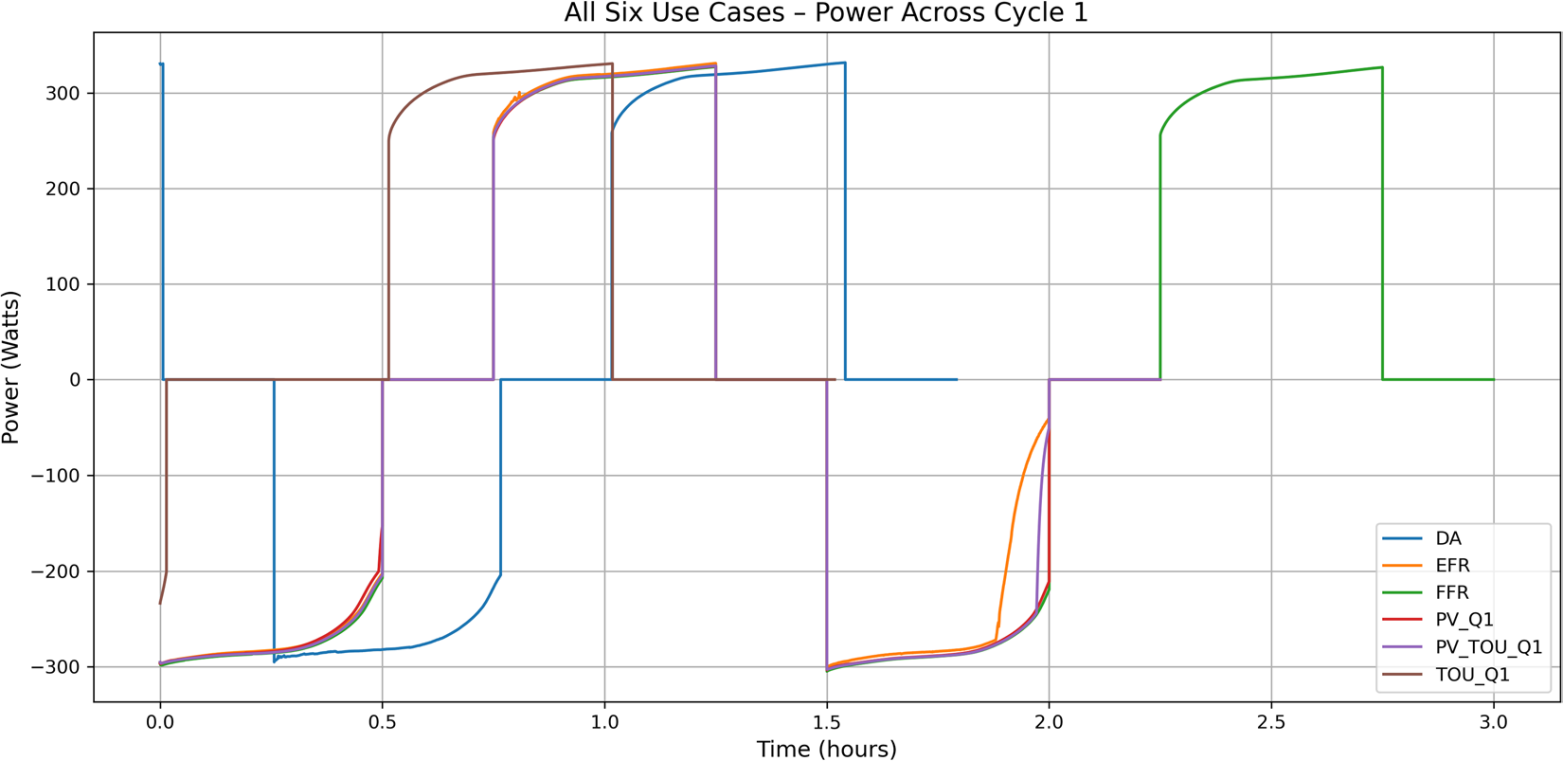
Power against Time plots for all six use cases.

In all six use cases, the first cycle was programmed to begin with a capacity test designed to establish an initial reference for battery performance. This test typically involves charging the battery module to an upper voltage threshold, followed by a controlled discharge, allowing for capacity measurement. Figure 6 illustrates this behaviour by plotting the Power against Time profiles for all six use cases for cycle 1.

**Fig. 7:**
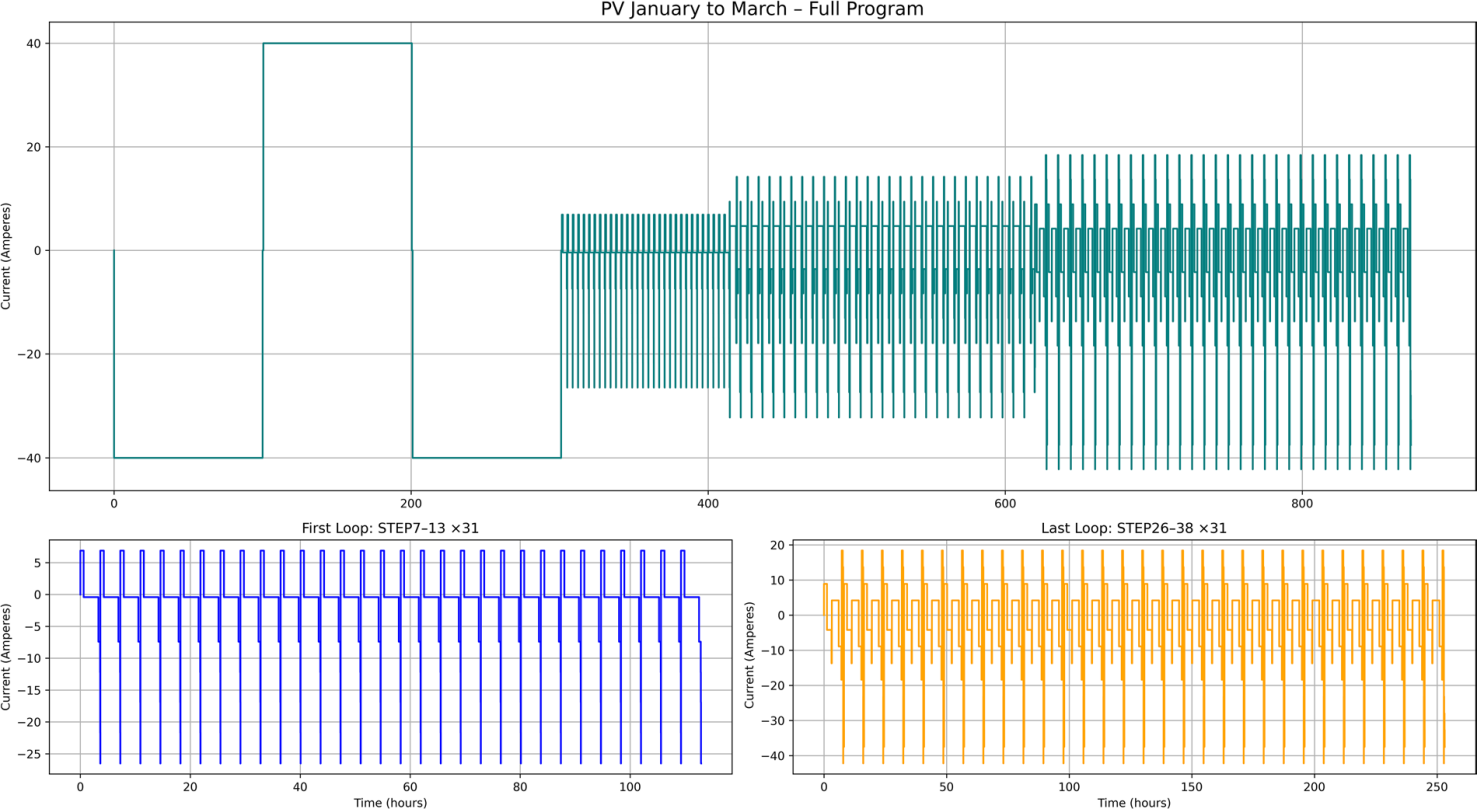
PV use case – Current versus time for January to March testing.

**Fig. 8:**
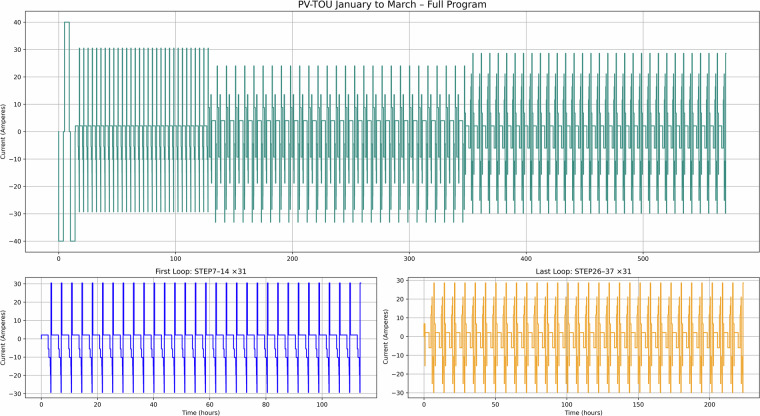
PV-TOU use case – Current versus time for January to March testing.

**Fig. 9:**
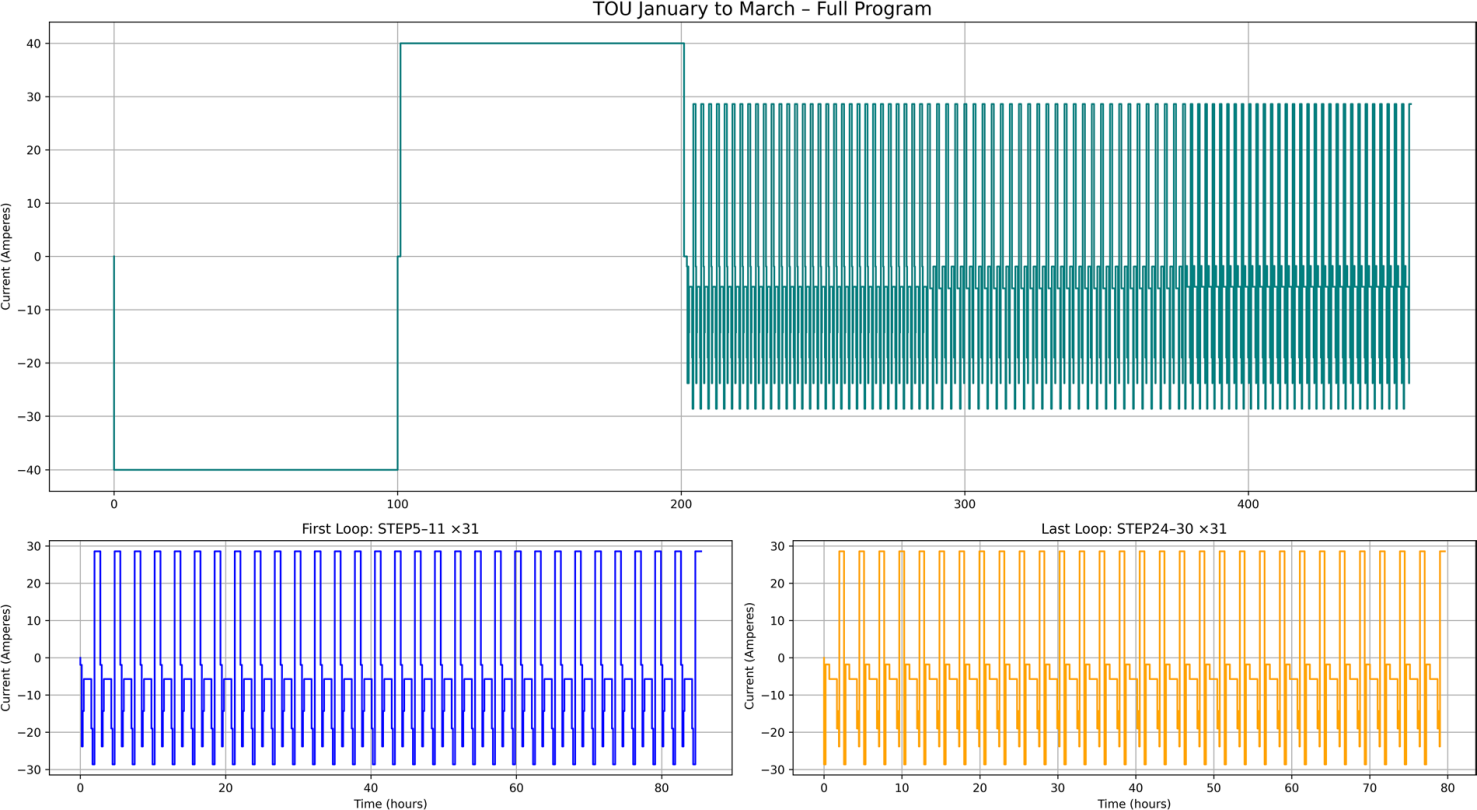
TOU use case – Current versus time for January to March testing.

**Fig. 10:**
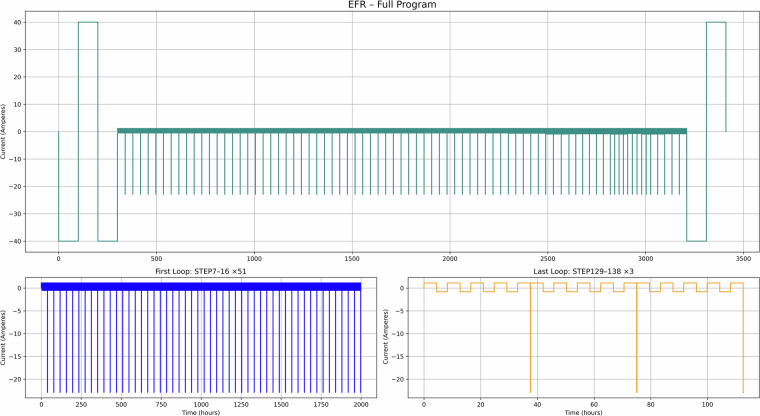
EFR use case – Current versus time for the full test recipe.

**Fig. 11:**
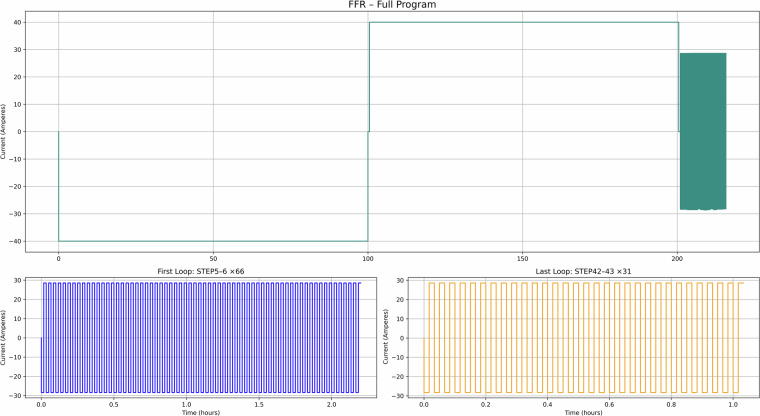
FFR use case – Current versus time for the full test recipe.

**Fig. 12:**
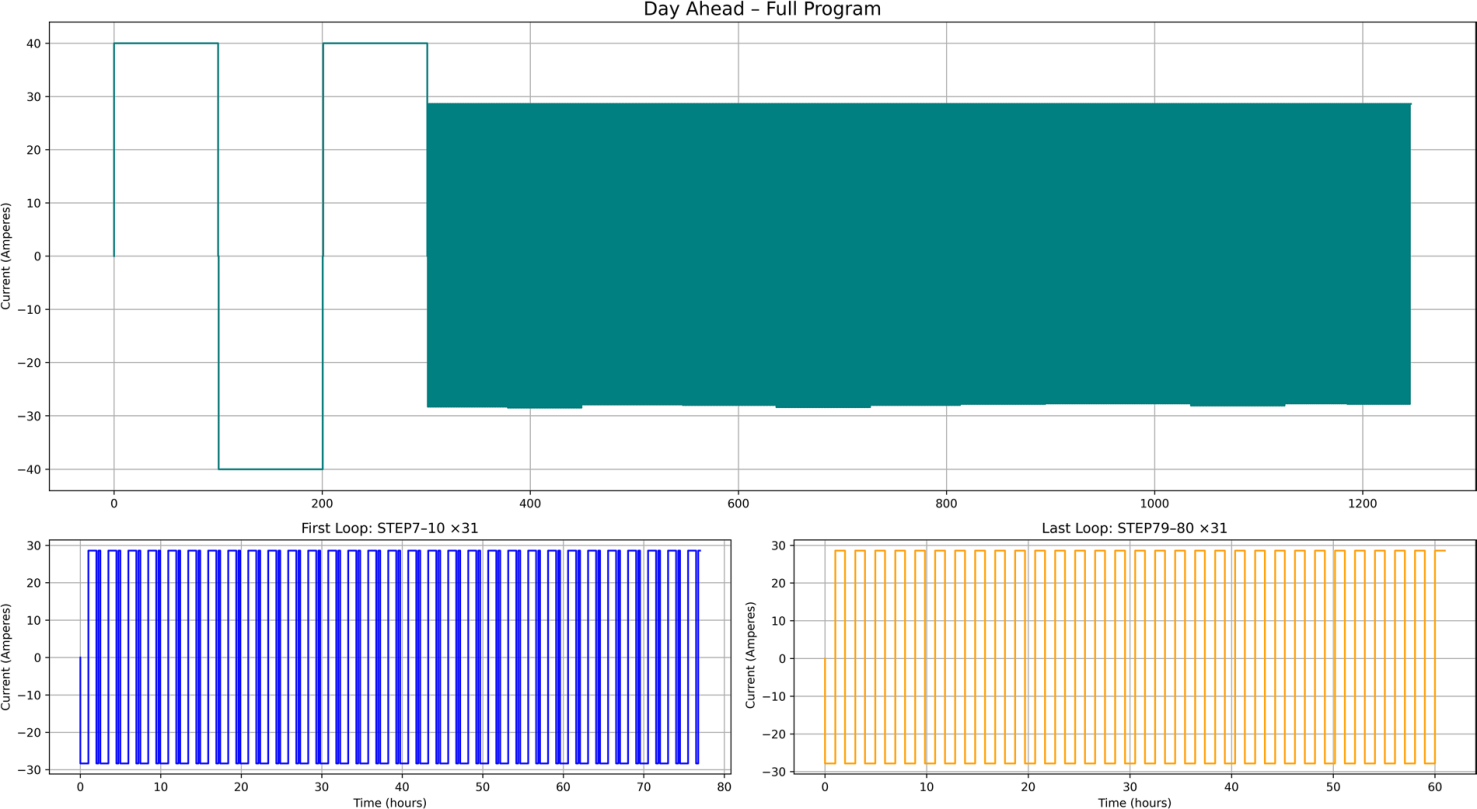
Day ahead use case – Current versus time for the full test recipe.

Additionally, Figs. 7 to 12 present the current-time profiles for each application based on their respective complete sweat test recipes. For PV, PV-TOU, and TOU, the figures show the entire January to March testing recipes, while for Day Ahead, FFR, and EFR, the full test recipes are shown in their entirety. Each figure consists of three panels: the top panel visualises the full current profile, whilst the bottom left and right panels highlight the first and final looping segments, respectively. These plots are designed to reflect realistic daily cycling behaviour for each application, enabling their direct use in long-term degradation testing. These can then be applied in long-term testing scenarios to evaluate battery performance and degradation across second-life use cases.

Figure 7 corresponds to use case 1 (PV with FIT maximisation), where excess solar energy is stored and used later in the day. This dataset is valuable for modelling solar self-consumption in domestic settings. Figure 8 shows use case 2 (PV with FIT and time-of-use tariff), which demonstrates how batteries can be used to optimise both renewable generation and electricity pricing, providing insight into dual-incentive optimisation strategies. Figure 9 covers used case 3 (TOU tariff only, no PV), capturing charge-discharge cycles based solely on tariff periods. This can be useful for analysing battery performance in non-solar, tariff-driven applications.

Figure 10 illustrates use case 4 (FFR), showing short one-minute cycling patterns (in line with the National Grid data) aligned with grid frequency events, which can be used for understanding degradation under high-frequency response services. Figure 11 presents use case 5 (EFR), where dynamic frequency response leads to repeated deep discharges as the system maintains a nominal state of charge. Finally, Fig. 12 depicts use case 6 (Day Ahead market), which models energy trading based on price signals, supporting the analysis of SLB suitability for energy arbitrage in market-based applications^10^.

Taken together, these figures highlight the diversity of cycling behaviours represented in the dataset. The PV and TOU-based use cases (Figs. 7–9) show structured daily profiles with moderate depth-of-discharge variations, reflecting energy use in residential settings. In contrast, the grid response use cases (Figs. 10–11) exhibit high-frequency cycling with minimal rest periods, which may impose greater mechanical and thermal stress on the cells. The Day Ahead profile (Fig. 12) displays more irregular cycling, driven by market volatility, with both shallow and deep discharges occurring intermittently. This range of cycling patterns demonstrates the dataset’s utility for exploring how SLBs perform under varied operational stressors.

## Data Extraction and Processing

**Fig. 13:**
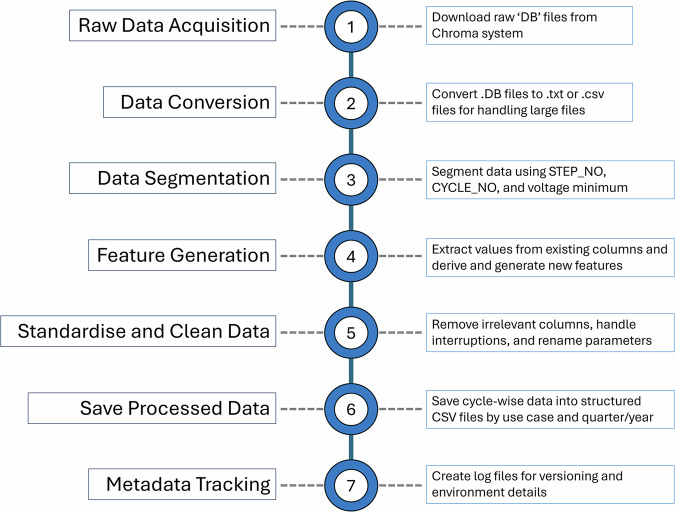
Overview of the data extraction and processing workflow.

To illustrate the complete processing workflow, Fig. 13 below provides a visual overview of full data extraction and processing workflow. The diagram summarises the end-to-end process, from acquiring raw.DB files through to saving the processed data and generating metadata. This flowchart serves as a high-level map, helping reader follow the structure of the following sections, which describe each stage in greater detail.

The raw data were downloaded from the PC connected to the Chroma battery tester as.DB files and were converted into.txt format as some files exceeded the.csv size limit. Converting these files into.txt format allowed the dataset to then be processed and cleaned. The data processing and cleaning were performed using Python (version 3.12.4) within Jupyter Notebook (version 7.0.8). The primary aim of this processing was to restructure the continuous raw data into smaller, individual cycles, making it easier to interpret, analyse, and apply to different tasks such as performance evaluation, degradation analysis and other tasks. Although the data remains unfiltered, and retains its raw measurement values, key features were also further extracted from existing features to organise the data into a more usable format. The key processing steps included:

## Segmentation of Testing Profile

The ‘STEP_NO’ column was used to track the sequence of charge-discharge steps over time. Some steps exhibited fluctuations between alternating short charge and discharge periods, requiring separation of cycles that consisted of repeated charge-discharge pulses from those representing longer, continuous test sequences. The STEP_NO and CYCLE_NO columns were analysed to categorize and extract structured charge-discharge periods. To define when a complete cycle ended, segmentation was performed based on the first detected minimum voltage point. This ensured that each recorded cycle captured both the initial charge and discharge behaviour, as well as the stabilization phase following voltage drops.

The dataset contained multiple experimental parameters stored within the EX_PARAMS_NAME_RECORD and EX_PARAMS_VALUE_RECORD columns. These parameters were extracted, standardized, and renamed for consistency. Some parameters included maximum and minimum voltage, average voltage, voltage difference, and ambient temperature. This transformation enabled a structured and uniform representation of additional test conditions.

## Data Cleaning

Empty columns, columns with NaN values, and specific unwanted columns were removed to streamline the dataset. The following columns were excluded from the dataset as they provided no meaningful insights for analysis and were largely empty across all files: Removing these columns had no impact on the integrity or usefulness of the dataset.

- ‘DETAILCORRECTIONTIME’, ‘EXTCORRECTIONTIME’, ‘CORRECTIONFFSET’, ‘EXTREALTIME’
- ‘RECOVERY_CYCLE1_NO’, ‘EX_PARAMS_NAME’, ‘EX_PARAMS_VALUE’
- ‘REMARK’, ‘DESCRIPTION’, ‘CYCLE3_NO’, ‘CYCLE4_NO’, ‘CYCLE5_NO’
- ‘RECOVERY_CYCLE2_NO’, ‘RECOVERY_CYCLE3_NO’, ‘RECOVERY_CYCLE4_NO’, ‘RECOVERY_CYCLE5_NO’

## Final Cycle Processing

The cleaned and segmented cycles were saved as individual.csv files. Each file included time-series data for the parameters defined in below. A feature column labelled ‘COMBINED’ was also added to indicate whether a given cycle had been merged from multiple datasets due to interruptions or restarts during long-term cycling tests.

## Defined Parameters

The dataset includes a wide range of parameters, and Tables 3 to 5 below defines and describes all of the parameters available in the dataset. Some parameters, such as maximum voltage, minimum voltage, average voltage, voltage difference, max position, and min position, are not always present in every dataset. These parameters are extracted from the EX_PARAMS_NAME_RECORD and EX_PARAMS_VALUE_RECORD fields, but their availability depends on whether the experimental data was recorded during that specific test cycle. If these fields are missing or incomplete, the corresponding parameters cannot be extracted.

**Table 3:** Description of the measured electrical parameters in the dataset (raw output).

Parameter	Description	Source
POWER	The measured power (W) during the cycle.	Raw
VOLTAGE	The measured voltage (V) of the module at the given time.	Raw
CURRENT	The measured current (A) flowing through the battery module.	Raw
CAPACITY	The accumulated charge (Ah) stored during the test.	Raw
KWH	The energy throughput (kWh) recorded during the test.	Raw
TOTAL CAPACITY	The total charge (Ah) processed by the battery over the entire cycle.	Raw
CHARGE CAPACITY	The charge capacity (Ah) recorded during a charging step.	Raw
DISCHARGE CAPACITY	The discharge capacity (Ah) recorded during a discharging step.	Raw

**Table 4:** Description of the raw system and metadata parameters recorded in the dataset (raw output).

Parameter	Description	Source
STEP IDX	The index of the current step in the cycling sequence.	Raw
PATTERN STEP NO	The predefined step number within the cycling pattern.	Raw
MODE	The operation mode of the battery cycler (running or stopped).	Raw
STEP NO	The unique identifier for a step within a given cycle.	Raw
CYCLE2 NO	A sub cycle numbering system for tracking test cycles.	Raw
CYCLE NO	A second sub cycle numbering system for tracking test cycles.	Raw
TEST TIME	The timestamp of the Chroma 17020.	Raw
TEST TIME ID	The elapsed test time from the start of the cycling process (centi-seconds – cs)	Raw
REAL TIME	The timestamp of the PC.	Raw
REAL TIME ID	The elapsed test time from the start of the cycling process on the PC (cs).	Raw
STEP DWELL TIME	The duration spent in a particular step (cs).	Raw
STEP MODE	Defines the specific operational mode of the battery module in a given step. This is an index value ranging from 0 to 3: 0 corresponds to rest, 1 represents a pause in the program, 2 denotes (CC-CV) charge, and 3 represents (CC) discharge.	Raw
STATUS IDX	An index value indicating the operational status of the battery module during testing.	Raw
EX PARAMS NAME RECORD	A record of additional experimental parameters stored as a text field.	Raw
EX PARAMS VALUE RECORD	The corresponding values for the additional experimental parameters.	Raw
RECORD ID	A unique identified for each recorded data entry.	Raw
DETAIL CORRECTION TIME	A timestamp or correction applied to the test data.	Raw
CUMULATIVE TIME	The total accumulated time (s) since the start of the test.	Raw

**Table 5:** Description of the generated and derived parameters in the dataset.

TIME BEFORE MIN	The time recorded before reaching the minimum voltage point.	Generated
TIME AFTER MIN	The time recorded after reaching the minimum voltage point.	Generated
PERIOD	The identified period within the test cycle – before or after the minimum voltage point.	Generated
COMBINED	A flag indicating whether a cycle was merged from multiple test segments.	Derived
CELL1	The voltage (V) of the first cell in the battery module.	Derived
CELL 2	The voltage (V) of the second cell in the battery module.	Derived
AMBIENT TEMPERATURE	The temperature (°C) of the surrounding environment during testing.	Derived
MAX VOLTAGE	The highest recorded voltage (V) during the cycle.	Derived
MIN VOLTAGE	The lowest recorded voltage (V) during the cycle.	Derived
AVG VOLTAGE	The average voltage (V) over the duration of the cycle.	Derived
VOLTAGE DIFFERENCE	The difference between the maximum and minimum voltage recorded during the cycle.	Derived
MAX POSITION	The time index or step where the maximum voltage was observed.	Derived
MIN POSITION	The time index or step where the minimum voltage was observed.	Derived

Throughout the cycling, there were some cases where the test cycles were restarted due to undervoltage protection being activated, potentially introducing additional aging effects not explicitly visible in the dataset. Testing was also paused on weekends and long holidays. To address the resulting data gaps and maintain a continuous representation of cycling, some datasets were manually merged. A feature labelled “COMBINED” (yes/no) has been added to indicate whether multiple files were combined in this way. However, it is important to note that while this merging helps to fix the discontinuities, it assumes that no significant changes occurred during these pauses, and there may still be residual inconsistencies.

The ‘Source’ column in Tables 3 to 5 indicates whether a parameter is directly from the raw Chroma output (raw), calculated or extracted from the existing raw fields (Derived), or newly created during the processing phase (Generated) to support further analysis or feature engineering.

Following the extraction and processing of the raw .DB files, the dataset sizes were compared before and after the cleaning and transformation steps. The initial sizes represent the raw extracted and converted .CSV files, while the processed sizes reflect changes due to the removal of irrelevant columns and the generation of additional cycle-level features. In some cases, the data sizes decreased due to the removal of empty or redundant fields from the raw Chroma output. However, in other cases, such as PV-TOU, TOU, and FFR, the dataset size increased slightly after processing. This increasing is primarily due to the successful extraction of additional parameters from the ‘EX_PARAMS_NAME_RECORD’ and ‘EX_PARAMS_VALUE_RECORD’ fields. When present, these fields contain embedded experimental metadata, which was parsed and used to generate new columns (e.g., MAX VOLTAGE, MIN VOLTAGE, AVG VOLTAGE, etc.). Since the presence of this metadata varies across test cycles and use cases, the resulting file size changes depend on the availability of these values during each test cycle. Table 6 below summarises the change in file size for each use case in gigabytes (GB), along with the corresponding percentage change.

**Table 6:** Change in dataset size before and after processing for each use case.

Use Case	Before (GB)	After (GB)	Percentage Change
1. PV	6.904	5.852	−15.24%
2. PV-TOU	5.170	5.650	+9.28%
3. TOU	4.490	4.860	+8.25%
4. EFR	8.180	5.750	−29.67%
5. FFR	5.450	5.530	+1.47%
6. Day Ahead	8.950	6.780	−24.25%

## Data Records

All data files associated with this study have been made publicly available through the Loughborough University Research Repository (LUPIN)^15^: 10.17028/rd.lboro.28732490.v2.

The dataset is provided as a compressed. zip archive, which contains separate folders for each of the six use cases: PV, PV-TOU, TOU, EFR, FFR, and Day Ahead. Each folder contains pre-processed cycle-level data in. csv or. txt format.

For the PV, PV-TOU, and TOU use cases, data is further organised by quarter. Within each use case folder, subfolders are labelled according to the actual test dates, using a “YYYY_Month_Month” format (e.g., 2022_April_June) to reflect the period during which testing was conducted. This allows users to easily identify and access data for specific time ranges within a given use case. The EFR, FFR, and Day Ahead use cases follow a year-long cycle format and are stored as single folders per use case, without further subdivision.

In addition to the main datasets a supplementary information folder is included, containing detailed test recipes used in the Chroma 17020 battery cycle for the six use cases. These documents provide cycle-by-cycle test descriptions for users who wish to reference or replicate specific testing protocols.

Two additional .txt files are included at the root level:

- Changelog.txt – summarises dataset updates and file additions, including date stamps and relevant notes.
- Requirements.txt – lists the python package dependencies used in the data extraction and processing scripts.

## Technical Validation

The dataset has been validated by comparing reconstructed waveforms with original charge/discharge profiles, performing standard error checks to identify inconsistencies, preprocessing to align time-series measurements and remove anomalies, and conducting internal consistency checks to ensure accurate representation of battery behaviour.

### Known incidents during testing

Several factors impacted the consistency of the dataset. This included a change in testing location of the batteries and subsequent storage over the period of the move. Test restrictions during COVID and the requirement to check calibration of the sensors approximately mid-way through the testing. Capacity tests were attempted at regular intervals, but these did not always register correctly as the CC-CV charge that precedes a full discharge did not always complete the CV part due to issues with midpoint protection^11^. As a result, only the start and end capacity values are reported here as these were reliably obtained. Additionally, one of the PV tests was conducted under an incorrect testing profile for an unspecified duration. This discrepancy was not actively tracked at the time and may have introduced variability or inconsistencies in the recorded charge-discharge behaviour for the particular use case.

The initial start and end capacity values for each use case are provided in Table 7 below.

**Table 7:** Start and end capacity values for each use case.

Use Case	Initial Capacity (Ah)	Final Capacity (Ah)
1: PV	45.37	41.64
2: PV-TOU	44.39	20.62
3: TOU	45.21	42.92
4: FFR	44.60	38.18
5: EFR	44.51	39.60
6: Day Ahead	44.11	33.16

## Usage Notes

This dataset is intended for research applications in battery state of health prediction, aging analysis, performance benchmarking, and energy market research. It supports the development of machine learning models for SOH estimation, enables detailed tracking of degradation trends over time, provides a reference for comparing battery degradation across different cycling conditions, and is useful for evaluating the economic feasibility of second-life batteries in various applications. The dataset does not include sophisticated demand-side management or electric vehicle fast charging applications, as these were not market-ready at the time of testing. Microgrid functionality and large-scale peak load shaving were also not included due to the absence of relevant market mechanisms.

## Data Availability

Example Jupyter Notebook scripts for converting the raw.DB data into.csv or.txt formats are freely available in the online repository at 10.17028/rd.lboro.28732490.v2^15^. These scripts serve as the starting point for data extraction and enable users to work with files that exceed.csv size limits. The repository also includes processing scripts tailored to each use case – separate scripts are provided to segment the PV-TOU, TOU, EFR and Day Ahead data into individual cycles, the PV dataset into smaller cycles, and the FFR dataset into one-minute response cycles. An additional script is provided for feature engineering, which derives and generates new columns to support further analysis. The required and optional Python libraries are listed in the ‘requirements.txt’ file in the online repository.
